# Delayed enterocutaneous fistula formation secondary to an inverted non-absorbable suture post midline laparotomy closure

**DOI:** 10.1016/j.ijscr.2020.05.035

**Published:** 2020-06-01

**Authors:** Danylo Yershov, Paul Murphy, Henry Ferguson

**Affiliations:** South Warwickshire NHS Foundation Trust, Lakin Rd, Warwick, CV34 5BW, United Kingdom

**Keywords:** Enterocutaneous fistula, Laparotomy, Sutures, Postoperative complications

## Abstract

**Introduction:**

Enterocutaneous fistula is a well recognised complication of intra-abdominal surgery. Postoperatively, it may occur due to a small bowel injury or an anastomotic leak and usually presents within days or weeks after surgery.

**Methods:**

We present a case report. Information was obtained through a retrospective review of the notes, clinic letters, images and the histology report.

**Results:**

We present a case of a patient who developed an enterocutaneous fistula 34 years after panproctocolectomy for ulcerative colitis. Imaging was consistent with an enterocutaneous fistula arising from mid-small bowel. A laparotomy and small bowel resection was performed. The enterocutaneous fistula occurred due to an inverted non-absorbable suture post midline laparotomy closure.

**Conclusion:**

Suture-related enterocutaneous fistula can be a rare delayed complication of a midline laparotomy closure when a knot is inverted intra-peritoneally. Consideration should be given to either leaving a knot in the subcutaneous fat tissue or feeding it between the rectus sheath and the suture bites.

## Highlights

• Such a delayed manifestation of enterocutaneous fistula secondary to a midline closure suture has not been previously described.
• We recommend to investigate such patients with a contrast CT scan to visualise the anatomy and an MRI scan to exclude Crohn's disease.
• The learning point is that inversion of the suture intra-peritoneally post midline laparotomy closure should be avoided.

## 1 Introduction

Enterocuntaneous fistula (ECF) is a well recognised complication of intra-abdominal surgery. Postoperatively, it may occur due to small bowel injury or anastomotic leak in both laparoscopic and open procedures. ECF usually presents within days or weeks after surgery. We present a case of ECF secondary to a non-absorbable suture which developed many years after the index procedure. The work has been reported in line with the SCARE criteria [1].

## 2 Case report

**Fig. 1:**
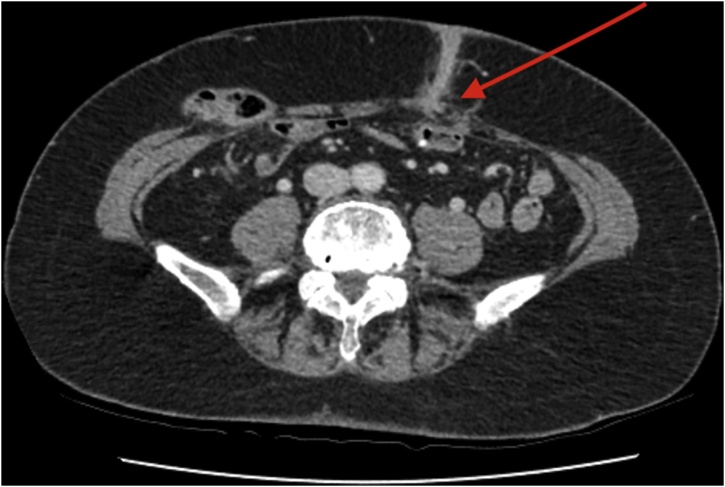
Contrast CT image of the ECF in the axial plane.

**Fig. 2:**
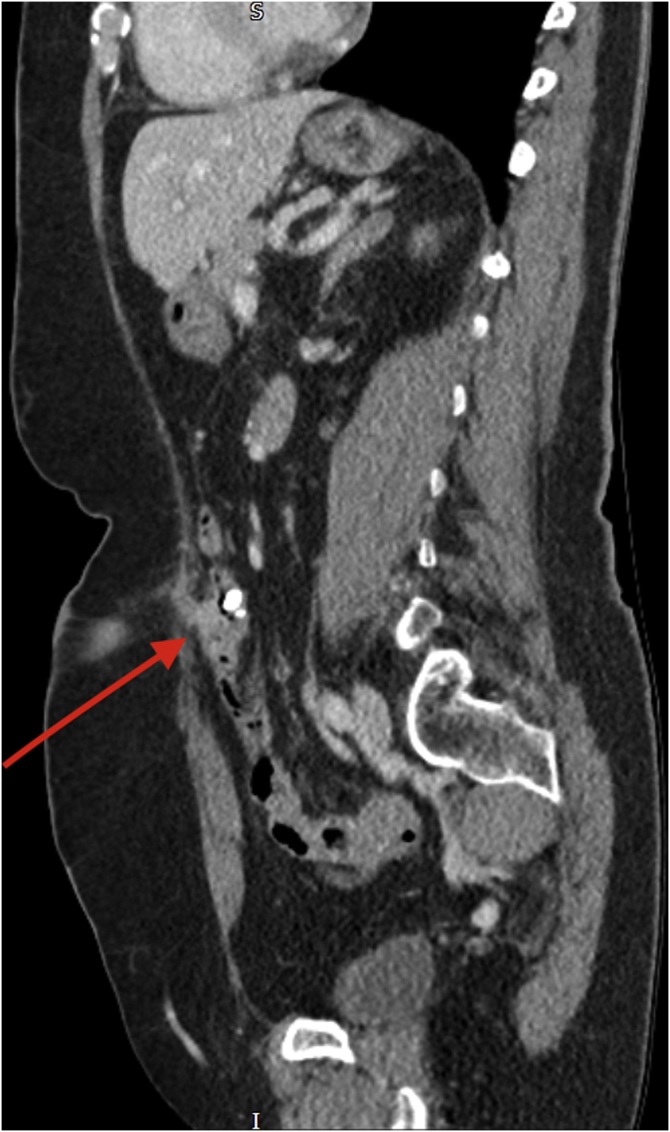
Contrast CT image of the ECF in the sagittal plane.

**Fig. 3:**
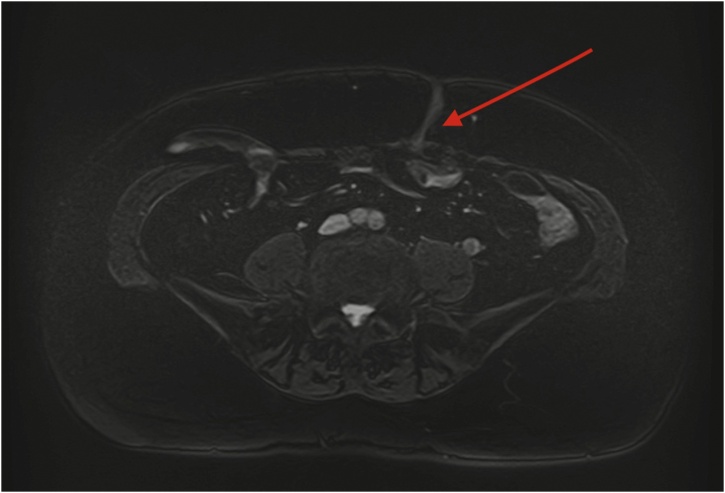
MRI image of the ECF in the axial plane.

We report a case of a 65 years old patient who presented to a colorectal clinic with an inflamed area discharging pus through the central portion of the midline scar 34 years after panproctocolectomy for ulcerative colitis. Contrast CT of abdomen and pelvis (CT AP) showed a tract leading from the skin surface to the mid small bowel through the left rectus abdominis muscle (Figs. 1, 2). MRI confirmed the presence of an ECF (Fig. 3), and refuted Crohn’s disease.

**Fig. 4:**
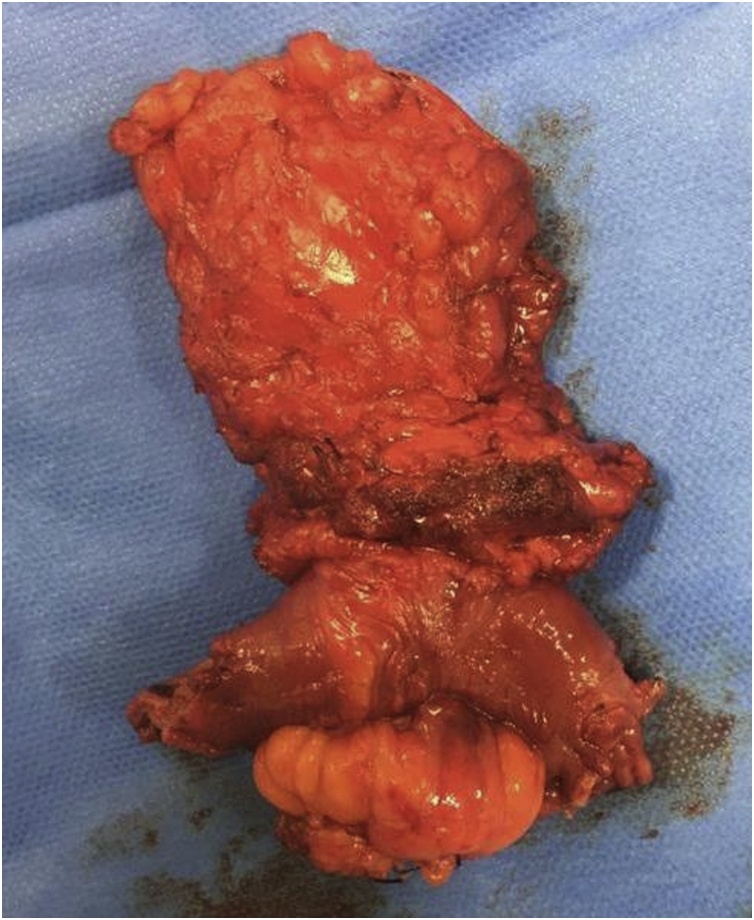
Specimen. Top to bottom: subcutaneous fat tissue, muscles of the anterior abdomen, small bowel, mesentery.

**Fig. 5:**
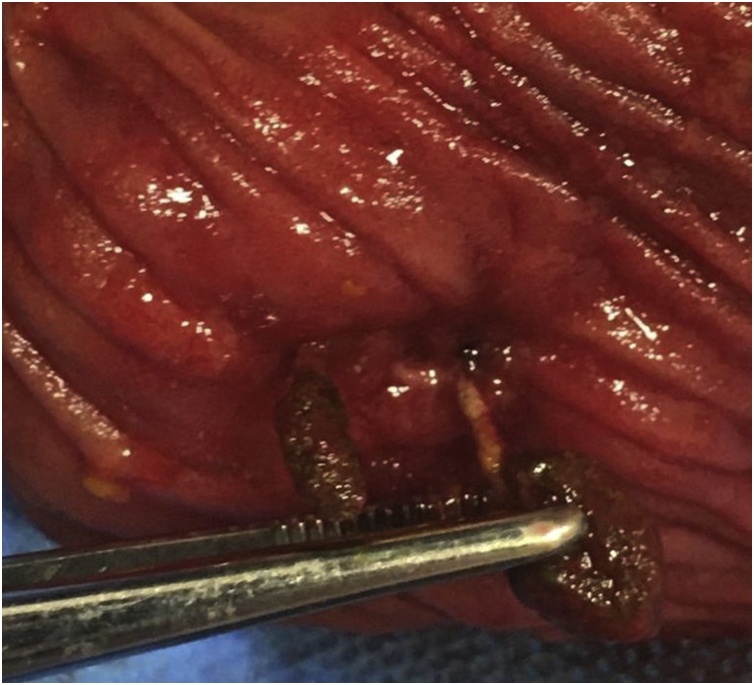
Intra-luminal view of the resected small bowel showing two ends of the stitch with attached fecaliths.

A laparotomy was performed through an elliptical excision of the fistula tract. An en bloc excision of the small bowel, muscle and skin layer was performed with side-to-side stapled anastomosis (Fig. 4). On opening of the specimen, two fecoliths arising from suture material were noted (Fig. 5). Interestingly, both ends of the suture were intra-luminal, but the knot remained outside the bowel, indicating the cut ends might have been left long.

The patient recovered well and was discharged home on post-operative day (PoD) 5.

Histology showed two pedunculated calcified projections 4 × 3 × 7 mm and 7 × 5 × 10 mm around a thick, blue suture material within the bowel segment. Microscopically, granulation tissue with giant cells lined tract extending from the small bowel lumen to the skin surface was reported. The findings were consistent with the suture-related ECF.

The patient re-attended with a wound infection on PoD 10. CT AP excluded an anastomotic leak, but the patient required antibiotics and drainage of the wound under general anaesthetic with satisfactory wound healing by secondary intention.

## 3 Discussion

Fistula formation secondary to a foreign material is not an uncommon surgical complication. Sigmoid and appendiceal fistulae were reported 7 years after a laparoscopic inguinal hernia repair with mesh [2]. Suzuki et al. reported a case of fistula formation into the appendix and the bladder 8 years after a Kugel inguinal hernia repair with mesh [3]. Enterovesical fistula 20 years after the Stamey procedure for incontinence was described [4]. Ileal fistula secondary to a retained surgical gauze 21 years after hysterectomy has previously been observed [5], with Yacoub et al. reported a case of ECF formed due to a spontaneous extrusion of an ingested wooden piece in a 2 year old boy [6]. However, such a delayed manifestation of ECF secondary to a midline closure suture has not been previously described.

Midline laparotomy closure is a well studied area. A number of articles comparing different suture materials and techniques are available. The well established 4:1 ratio of suture to wound length closure concept [7] is currently changing to a small bites technique [8]. On the other hand, there is little research into what to do with the final knot in the middle of the closed rectus sheath. The most common techniques are to leave the knot, feed it under the previous bites, or bury it intra-peritoneally either with another bite or with a clip. This latter technique may be prone to complications, because a blindly placed stitch may catch the bowel immediately or sharp material may erode through the bowel many years later, such as in this case.

## 4 Conclusion

Suture-related ECF can be a rare delayed complication of a midline laparotomy closure when a knot is inverted intra-peritoneally. Consideration should be given to either leaving a knot in the subcutaneous fat tissue or feeding it between the rectus sheath and the suture bites.

## Declaration of Competing Interest

None.

## Sources of funding

None.

## Ethical approval

Ethical approval has been exempted.

## Consent

Written informed consent was obtained from the patient for publication of this case report and accompanying images. A copy of the written consent is available for review by the Editor-in-Chief of this journal on request.

## Author contribution

**Danylo Yershov**: Conceptualization, Writing - Original Draft; **Paul Murphy**: Investigation; **Henry Ferguson**: Writing - Review & Editing, Supervision.

## Registration of research studies

N/A.

## Guarantor

Mr Henry Ferguson.

## Provenance and peer review

Not commissioned, externally peer-reviewed.
